# Micro- and nanoplastics reduce the phagocytosis and intracellular killing of *E. coli* by THP1-Blue™ NFκB monocytes

**DOI:** 10.1007/s15010-025-02565-1

**Published:** 2025-05-26

**Authors:** Florian Edbauer, Hans-Christoph Ludwig, Marie Julia Moritz, Roland Nau, Jana Seele

**Affiliations:** 1 Department of Neuropathology, University Medical Center Göttingen, Georg-August-University Göttingen, Göttingen, Germany; 2 Department of Geriatrics, Evangelisches Krankenhaus Göttingen-Weende, Göttingen, Germany; 3 Department of Neuropathology, University Medical Center Göttingen, Göttingen, Germany Robert-Koch-Strasse 40, 37075

**Keywords:** Microplastic, Nanoplastic, Human monocyte cell line, *Escherichia coli*, Phagocytosis, Bactericidal activity

## Abstract

**Purpose:**

Micro- and nanoplastic particles occur ubiquitously in the environment and have been detected in various organs in animals and humans. We studied, how micro- and nanoplastic influence phagocytosis and intracellular killing of live bacteria in human monocytes.

**Methods:**

Cells of the human reporter cell line THP1-Blue™ NFκB were pre-treated with different concentrations of micro- and nanoplastic (diameter 1 μm and 100 nm) and then incubated with *Escherichia coli* DH5α. Phagocytosis and intracellular killing was studied using an antibiotic protection assay. The activation of the NFκB promoter was quantified by measuring the production of alkaline phosphatase. Cytokines were measured by enzyme immunoassay. Cell viability was determined by trypan blue staining and lactate dehydrogenase measurement. Electron microscopic images were taken to localize micro- and nanoplastic.

**Results:**

Micro- and nanoplastic particles were rapidly internalized by monocytes. They reduced phagocytosis of *E. coli* in a concentration- and time-dependent manner. Exposure to micro- and nanoplastic also reduced the intracellular killing of bacteria in a concentration-dependent manner. Plain plastic particles did not induce NFκB synthesis and IL1β and IL6 release. At concentrations inhibiting phagocytosis, micro- and nanoplastic was not cytotoxic. Endotoxin stimulated phagocytosis of bacteria. High concentrations of plastic particles reduced the stimulatory effect of endotoxin on phagocytosis of bacteria, but not the effect on NFκB synthesis.

**Conclusion:**

Exposure to micro- and nanoplastic reduced the ability of phagocytes to internalize and kill bacteria. High plastic concentrations decreased the endotoxin-stimulated phagocytosis of bacteria. Hence, exposure to plastic particles may reduce the host`s immune defence against bacterial pathogens.

**Supplementary Information:**

The online version contains supplementary material available at 10.1007/s15010-025-02565-1.

## Introduction

Plastic is a heterogeneous group of synthetic or semisynthetic compounds with polymers as their main constituents. The first representatives of this group were already synthesized in the 19th century [1]. Originally, small amounts were produced. In recent decades, production grew exponentially. Most plastic materials are cheap, durable and can be easily formed without breaking. At present, estimates of the production of plastic per year range from 270 to more than 390 million tons [2, 3]. Humans are exposed to micro- and nanoplastic by air [4], fresh water [5], salt water [6], beverages and food [3, 7–10]. Plastics increasingly are a secret threat to human and planetary health. Plastics…”are responsible for grave harms to health, widespread environmental damage, great economic costs, and deep societal injustices. These harms are rapidly worsening….Global intervention against the plastic crisis is needed now because the costs of failure to act will be immense” [11].

Fragments of plastic with a diameter of 1–1000 μm are classified as microplastic, fragments with a diameter < 1 μm as nanoplastic [12]. Microplastic is generated either by mechanical stress (e.g. laundry of synthetic clothes, tyre and brake abrasion), deliberate addition of microplastic to commercial products (e.g. cosmetics) or (slower) by spontaneous degeneration of larger plastic items (e.g. bags, bottles, toys, fishing nets). Microplastic is further degraded into smaller pieces, until it becomes nanoplastic. Micro- and nanoplastic does not only pollute lakes and shallow parts of the sea, but also the deep sea and the sediments [6]. It is present in seafood [7, 13], in 81% of 159 tap water samples collected in different regions of the world [8] and in a variety of foods and beverages including beer [8, 9].

In recent years, research focused on the impact of micro- and nanoplastic on the animal life in salt- and freshwater, and the number of publications about the impact of micro- and nanoplastic on the immune response of mammals or mammalian cells is small. Micro- and nanoplastic is incorporated by eukaryotic cells. It can alter intracellular signal transduction and hereby can influence the immune response. Free radicals appear to be critically involved [2]. Polyethylene-, polypropylene- and polystyrene-containing microparticles degranulated granulocytes of Eurasian minnows (*Phoxinus phoxinus*) and mice and caused a release of neutrophil extracellular traps (NETs) which are able to immobilize pathogens and promote their phagocytosis [14, 15]. At high concentrations, phagocytosis of microplastic caused the death of immune cells in mussels [16]. Carboxylated polystyrene-containing particles (diameter approx. 1 μm) activated endothelial cells with subsequent adhesion of leukocytes in vitro and in vivo. In monocytes, they caused an increased production and release of pro-inflammatory cytokines [17]. In primary murine hepatocytes, cytotoxicity of plastic particles was size-dependent (larger were more cytotoxic than smaller particles), whereas very small nanoparticles (20 nm) were active in oxidizing lipids and disrupting mitochondrial function compared to larger particles [18]. Zebrafish exposed to a concentration of 500 µg/ml of polystyrene plastic particles (size 0.5–5 μm) displayed a reduced density of macrophages and an increased apoptosis of phagocytes. In murine RAW264.7 cells, both particle sizes induced the generation of reactive oxygen species and affected cellular metabolism [19].

Humans situated at the top of the food chain ingest high amounts of micro- and nanoplastic. The consequences of these particles on the function of our immune system, in particular on the ability to phagocytose and kill bacteria, are largely unknown. Here, the inhibitory effect of micro- and nanoplastic on the phagocytosis and intracellular killing of human immune cells is studied in vitro by means of THP1-Blue™ NFκB cells, a cell line derived from the peripheral blood of a 1-year-old boy with acute monocytic leukemia at his relapse in 1978 [20].

## Methods

### Transgenic human reporter cell line THP1-Blue™ NFκB

The transgenic human reporter cell line THP1-Blue™ NFκB (InvivoGen, San Diego, CA, USA) is derived from the human THP1 monocyte cell line by insertion of a NFκB-inducible secreted embryonic alkaline phosphatase (SEAP) reporter construct. The concentration of NFκB-induced SEAP in the cell culture supernatant is assessed with the reagent QUANTI-Blue™, developing a blue colour in response to the activity of SEAP. The cell line was cultured in RPMI-1640 supplemented with 2 mM L-glutamine, 25 mM HEPES, 10% heat-inactivated fetal calf serum (FCS), and penicillin-streptomycin (100 U/ml-100 µg/ml) plus 100 µg/ml Normocin™ (InvivoGen). After addition of plastic particles, cultures were placed on a laboratory shaker at 80 rotations per minute (RPM) in order to ensure homogeneous mixing of plastic particles with the cell culture medium.

### Micro- and nanoplastic particles

Spherical green-fluorescent polystyrene particles (Micromer-greenF: plain surface, size 100 nm, 1.9 × 10^12^ particles/mg, product code 29-00-102; size 1 μm, 1.9 × 10^9^ particles/mg, product code 29-00-103; NH_2_-coated surface, size 1 μm, 1.9 × 10^9^ particles/mg, product code 29-01-103; COOH-coated surface, size 1 μm, 1.9 × 10^9^ particles/mg, product code 29-02-103) were purchased from Micromod Partikeltechnologie, Rostock, Germany, as suspensions in water. Particles were diluted in Roswell Park Memorial Institute (RPMI) medium + 10% heat-inactivated FCS to the final concentrations in the individual experiments. For fluorescence microscopy, excitation wavelength was 475 nm, and emission wavelength was 510 nm.

### Light microscopy

After incubation of THP1-Blue™ NFκB monocytes with red-fluorescent *Escherichia (E.) coli* DH5α, a strain apathogenic for immunocompetent individuals transformed with plasmids encoding the red fluorescent protein DSRed (excitation wavelength 558 nm, emission wavelength 583 nm) [21, 22] (kind gift of Sven Hammerschmidt, University of Greifswald, Germany) and green-fluorescent microplastic, the contents of the wells (200 µl) were centrifuged at 300 x g for 5 min in a centrifuge (Megafuge 16R, Thermo Fisher Scientific, Waltham, MA, USA) on coverslips and fixed with 4% paraformaldehyde for 10 min. These coverslips were transferred to glass slides. Cells were embedded in Fluoromount G containing 4′,6-diamidino-2-phenylindole (DAPI) (Thermo Fisher Scientific, Waltham, MA, USA) to stain the nuclei of the phagocytes. Fluorescence and phase-contrast microscopy was performed on an Olympus BX51 Microscope (Olympus, Hamburg, Germany).

### Electron microscopy

After incubation with plastic particles, cells were fixated with 3% glutaraldehyde for 12 h. Post-fixation was performed with 1% OsO_4_. After dehydration with ascending ethanol series, cells were embedded in RenLam^TM^ without using propylene oxide. Ultrathin sections (70 nm) were cut with a diamond knife, stained with uranyl acetate and lead citrate, and then viewed with a Zeiss EM 10B transmission electron microscope (Zeiss, Oberkochen, Germany).

### Quantification of phagocytosis and intracellular killing

For the construction of concentration-response and time-response curves, THP1-Blue™ NFκB monocytes were pre-incubated with plastic particles of 1 μm and 100 nm in diameter at concentrations of 5 µg/ml, 25 µg/ml, 125 µg/ml, and 500 µg/ml for 24 h, 72 h and 216 h at 37 °C. Pre-incubation with 0.1 µg/ml LPS dissolved in RPMI with 10% FCS for 24 h in the absence of plastic particles served as positive control, and 24 h of an equal volume of RPMI + 10% FCS as negative control. After pre-incubation, THP1-Blue™ NFκB monocytes were co-incubated with *E. coli* DH5α for 30 min. Thereafter, extracellular bacteria were killed by gentamicin at a concentration of 100 µg/ml [23]. At the respective time point, phagocytes were washed with PBS and lysed by the addition of distilled water. The resulting suspension was quantitatively plated on blood agar plates. The *E. coli* colonies grown on the plates were counted after 16 h of incubation at 37 °C. For the assessment of the rate of phagocytosis, the number of intracellular bacteria was determined after 30 min of gentamicin treatment to kill extracellular adherent bacteria. For the assessment of intracellular killing, the number of intracellular bacteria was quantified at 30 and 180 min after co-incubation of phagocytes containing the phagocytosed *E. coli* DH5α with gentamicin. To ensure the inter-day comparability of individual experiments, the median number of phagocytosed bacteria in unstimulated control wells of each experiment after 30 min of phagocytosis followed by 30 min of gentamicin treatment to kill extracellularly situated bacteria was set as 100%, and the number of phagocytosed bacteria in each individual well was expressed as %. Intracellular killing was quantified by analysis of the decrease of the intracellular concentrations of *E. coli* between 30 min (= 100%) and 180 min of treatment with gentamicin in percent.

### Cytokines

Cytokines were measured in the supernatants after 24 h of co-incubation with plastic particles at different concentrations by enzyme immunoassay (ELISA MAX Standard Set Human IL-1β and Human IL-6, BioLegend™, San Diego, CA) according to the manufacturer`s instructions. Cytokines were also measured after pre-stimulation by 0.1 µg/ml LPS in the absence or presence of micro- or nanoparticles. The detection limit of the assays was 2 pg/ml for IL-1β and 7.8 pg/ml for IL-6.

### Cytotoxicity

The cytotoxic effect of plastic particles was quantified by lactate dehydrogenase (LDH) release by a coupled enzymatic assay (CytoTox 96™ Non-Radioactive Cytotoxicity Assay, Promega, Madison, WI, USA), which results in the conversion of iodonitro-tetrazolium violet (INT) into a red formazan product. The amount of color formed is proportional to the number of lysed cells. Absorbance at 490 nm was quantified in a 96-well plate reader (Bio-Rad iMark Microplate Reader, Bio-Rad Laboratories Feldkirchen, Germany). Since the half-life of LDH released from cells into the surrounding medium is approximately 9 h (Promega, manufacturer`s information), by this method cytotoxicity of micro- and nanoplastic was quantified at 24 h only.

The viability of the THP1-Blue™ NFκB monocytes was determined by the trypan blue exclusion method using a 1:5 dilution of 0.4% trypan blue (Sigma-Aldrich, Taufkirchen, Germany, product number T8154) in RPMI supplemented with FCS. After staining for 5 min, at least 100 cells were counted in a Neubauer hemocytometer, and the number of viable (unstained) cells was expressed in % of the total cell count.

### Statistics

Analysis was based on 12–24 independent measurements from experiments performed on at least 3 different days. Since data often were not normally distributed, data were expressed as medians and 25th /75th percentiles. Groups were compared by Kruskal-Wallis test followed by two-tailed Dunn’s multiple comparisons test to correct for repeated testing by means of GraphPad Prism Software (Version 6.0.; GraphPad, San Diego, CA, USA). P values < 0.05 were considered statistically significant.

## Results

### Concentration- and time-dependent inhibition of bacterial phagocytosis by micro- and nanoplastic

**Fig. 1:**
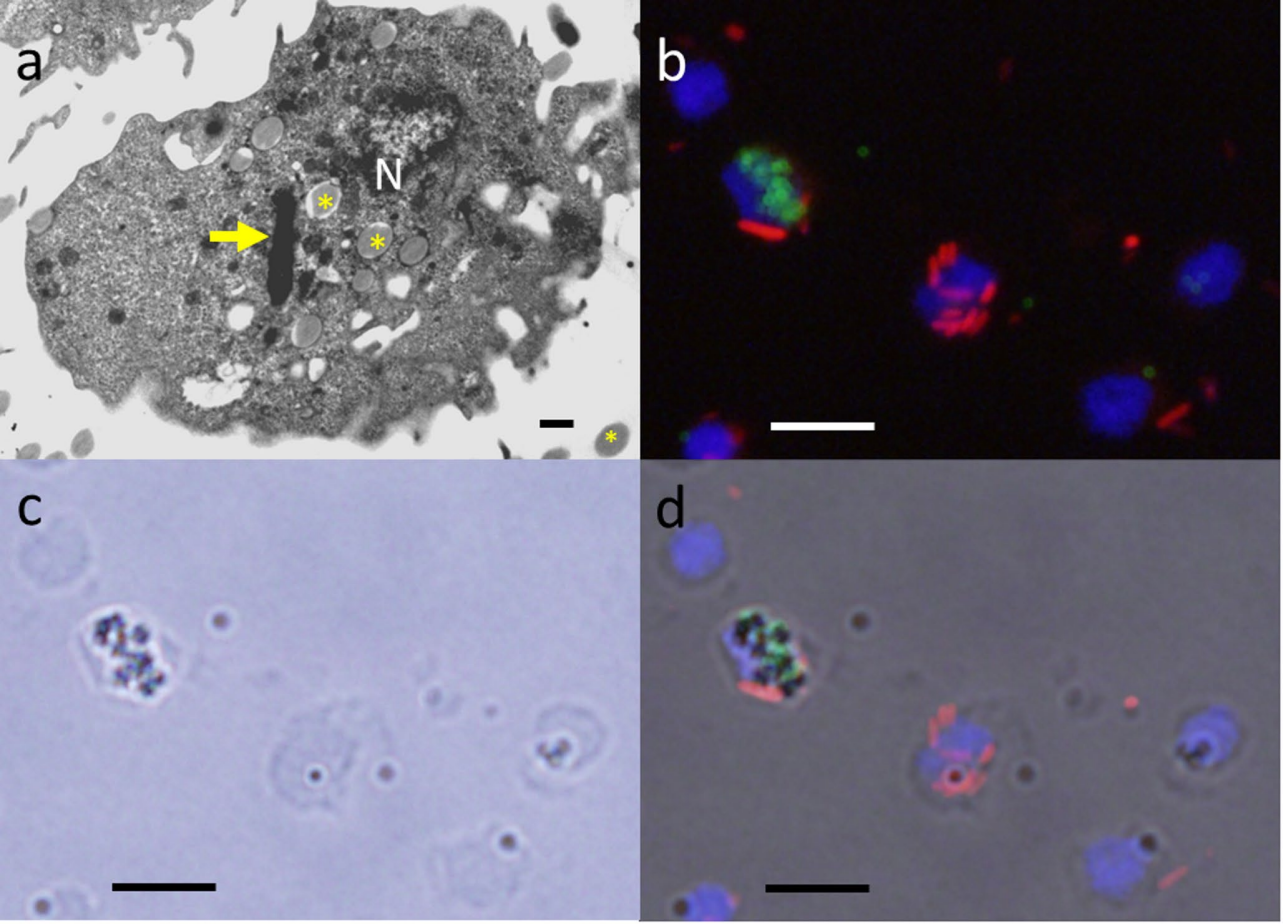
Microplastic and bacteria were rapidly ingested by phagocytes. (**a**) Electron microscopic image proving the intracellular presence of plastic particles (1 μm plain polystyrene microbeads) in THP1-Blue™ NFκB monocytes after pre-treatment with 500 µg/ml for 24 h. N: nucleus; asterisks: microplastic particles; arrow: phagocytosed *Escherichia coli*. **b-d**) Fluorescence microscopy of THP1-Blue™ NFκB cells after pre-incubation with 25 µg/ml microplastic (diameter 1 μm) for 24 h followed by incubation with *E. coli* DH5α for 60 min. (**b**) Immunofluorescence (green: microplastic; red: *E. coli*; blue: nuclei stained by 4′,6-diamidino-2-phenylindole [DAPI]), (**c**) phase contrast microscopy delineating the borders of the phagocytes, (**d**) merged image showing the predominantly intracellular location of plastic particles and bacteria. Space bar indicates 1 μm (**a**) and 15 μm (**b-d**)

**Fig. 2:**
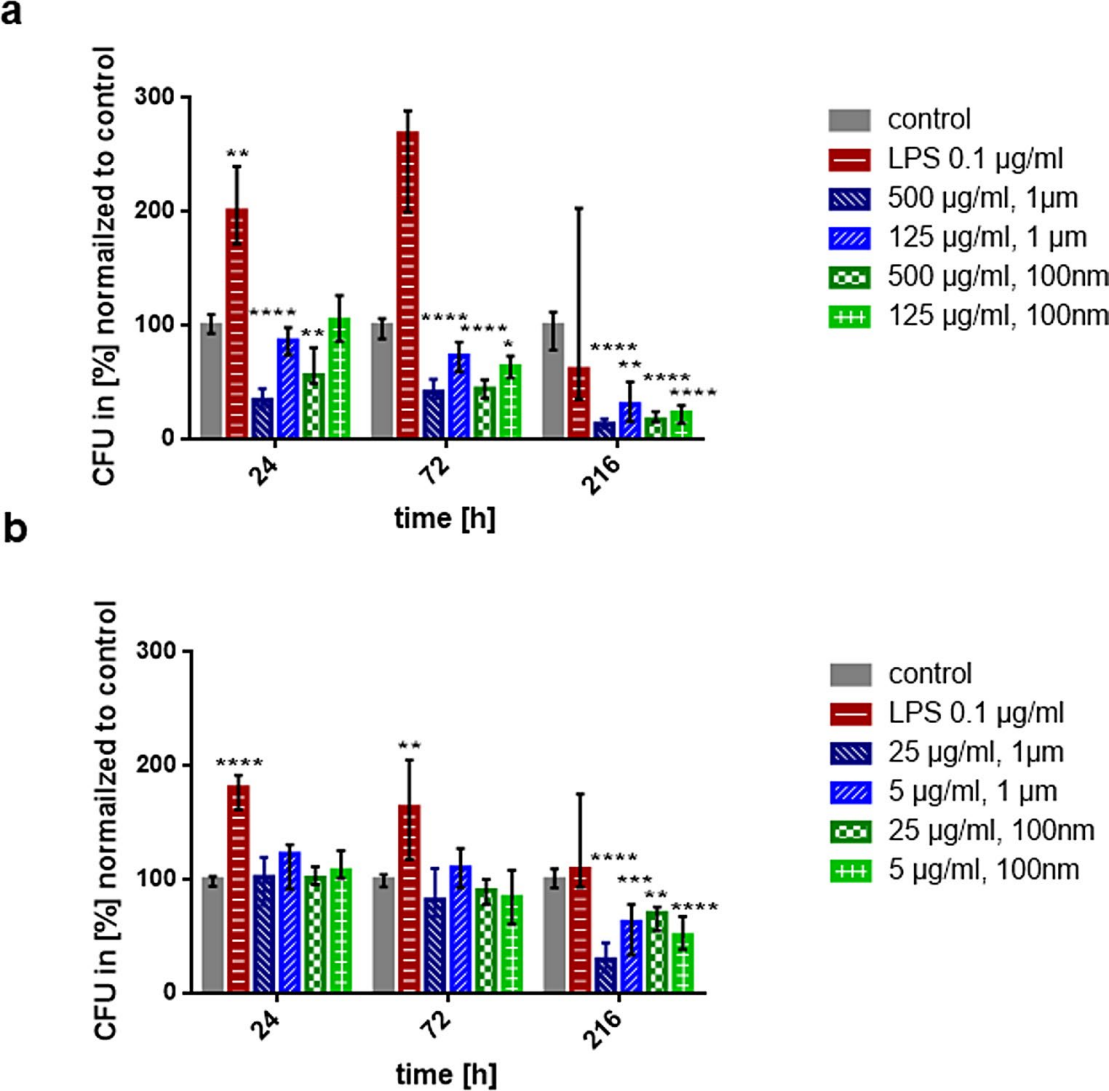
Micro- and nanoplastic at low concentrations inhibited phagocytosis of *E. coli* after prolonged exposure only, whereas plastic at high concentrations rapidly was anti-phagocytic. (**a**) Concentration-time relation of THP1-Blue™ NFκB phagocytosis of *E. coli* after pre-treatment with high concentrations of plastic particles of 2 diameters (100 nm and 1 μm, polystyrene, plain surface). High concentrations of micro- and nanoplastic already inhibited phagocytosis of bacteria at 24 h. (**b**) Concentration-time relation of phagocytosis of *E. coli* by THP1-Blue™ NFκB monocytes after pre-treatment with low concentrations of microplastic (1 μm) or nanoplastic (100 nm) particles (polystyrene, plain surface). Please note that low plastic concentrations affected bacterial phagocytosis after 216 h of pre-treatment. Statistical comparisons of all groups versus unstimulated controls; *n* = 18, columns represent medians, error bars indicate 25th and 75th percentiles; *p ≤ 0.05, **p ≤ 0.01, ***p ≤ 0.005, ****p ≤ 0.001

The effect of micro- and nanoplastic on the ability of the human THP1-Blue™ NFκB monocytic cell line to phagocytose unencapsulated *E. coli* Dh5α was studied from 24 to 216 h (Figs. 1a-d and 2a, amp and b). We observed a time- and concentration-dependent decrease of *E. coli* phagocytosis after 24, 72 and 216 h of preincubation with micro- and nanoplastic (500 µg/ml) (Fig. 2a & b). The highest concentration tested (500 µg/ml) of particles with a diameter of 1 μm or 100 nm inhibited phagocytosis of bacteria already at 24 h (Fig. 2a). At low concentrations of micro- and nanoplastic, the exposure for 24 h and 72 h did not influence phagocytosis. After 216 h of preincubation, a reduction in phagocytosis was observed with low plastic particle concentrations (5 and 25 µg/ml) as well (Fig. 2b). Electron microscopic images documented the intracellular presence of microplastic (Fig. 1a).

### Inhibition of the phagocytosis-stimulating activity of LPS by micro- and nanoplastic

**Fig. 3:**
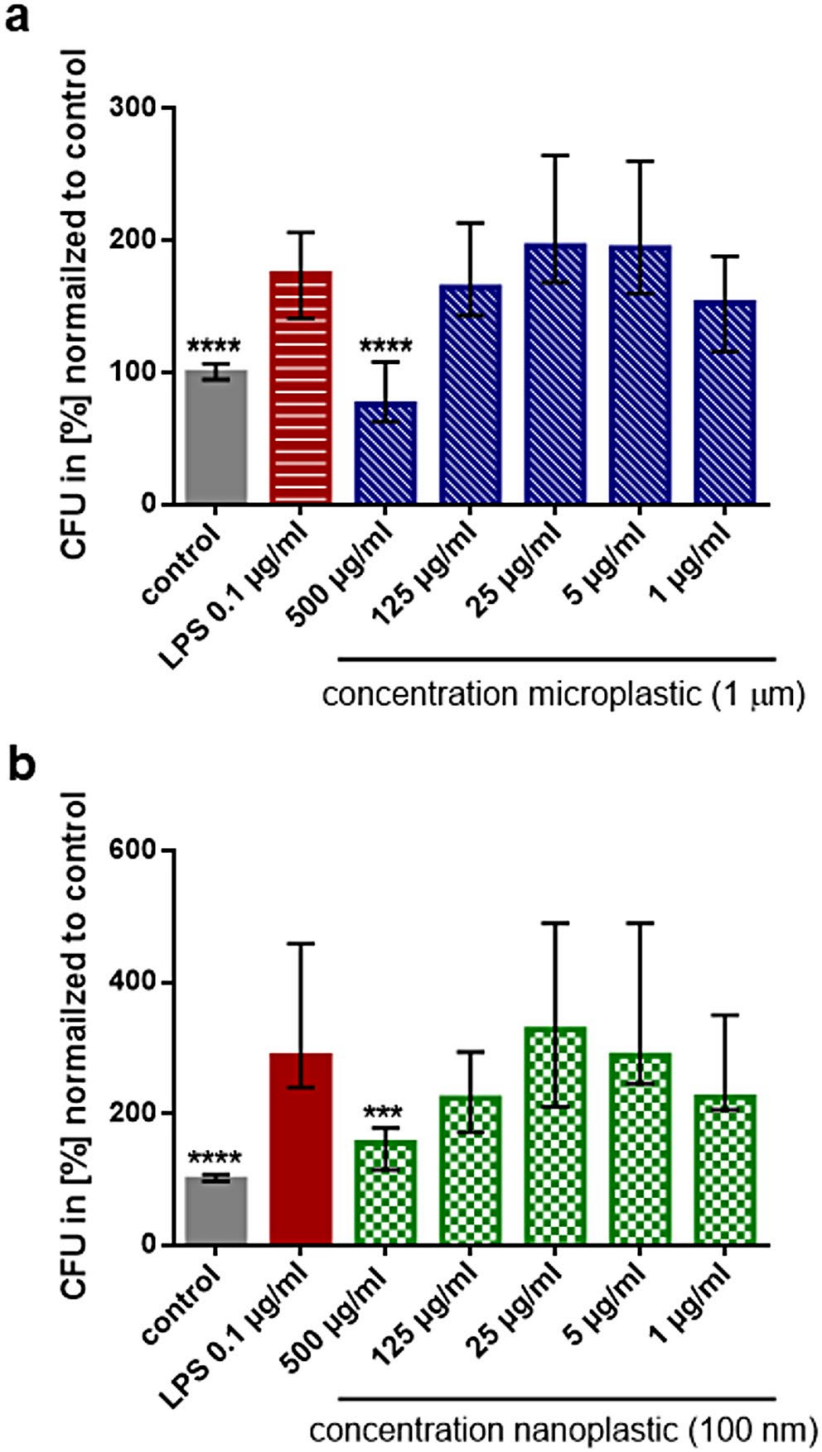
Plastic particles at high concentrations inhibited the immunostimulatory action of LPS on bacterial phagocytosis. (**a**) Microplastic, diameter 1 μm. (**b**) Nanoplastic, diameter 100 nm. Phagocytosis of *E. coli* by THP1-Blue™ NFκB monocytes after pre-treatment with different concentrations of microplastic particles together with 0.1 µg/ml LPS for 24 h. Please note that 500 µg/ml of plastic particles of both sizes inhibited the action of LPS at 24 h, whereas the other microplastic concentrations had no effect. Statistical comparisons of all groups versus LPS 0.1 µg/ml; *n* = 24, columns represent medians, error bars indicate 25th and 75th percentiles; *p ≤ 0.05, **p ≤ 0.01, ***p ≤ 0.005, ****p ≤ 0.001

In the absence of plastic particles, pre-stimulation for 24 h with LPS at a concentration of 0.1 µg/ml increased the phagocytosis of *E. coli* Dh5α by approx. 100%. Exposure to plastic particles with a diameter of 1 μm and 100 nm at the highest concentration of 500 µg/ml for 24 h inhibited the stimulatory action of LPS on phagocytosis of bacteria. Lower plastic particle concentrations did not influence the phagocytosis-stimulating activity of LPS at 24 h (Fig. 3). Microplastic (size 1 μm, plain surface, COOH- and NH_2_-coated surface) at all concentrations tested did not inhibit the LPS-induced activation of NFκB (Supplement, Figure S1).

### Inhibition of intracellular killing of bacteria by micro- and nanoplastic

**Fig. 4:**
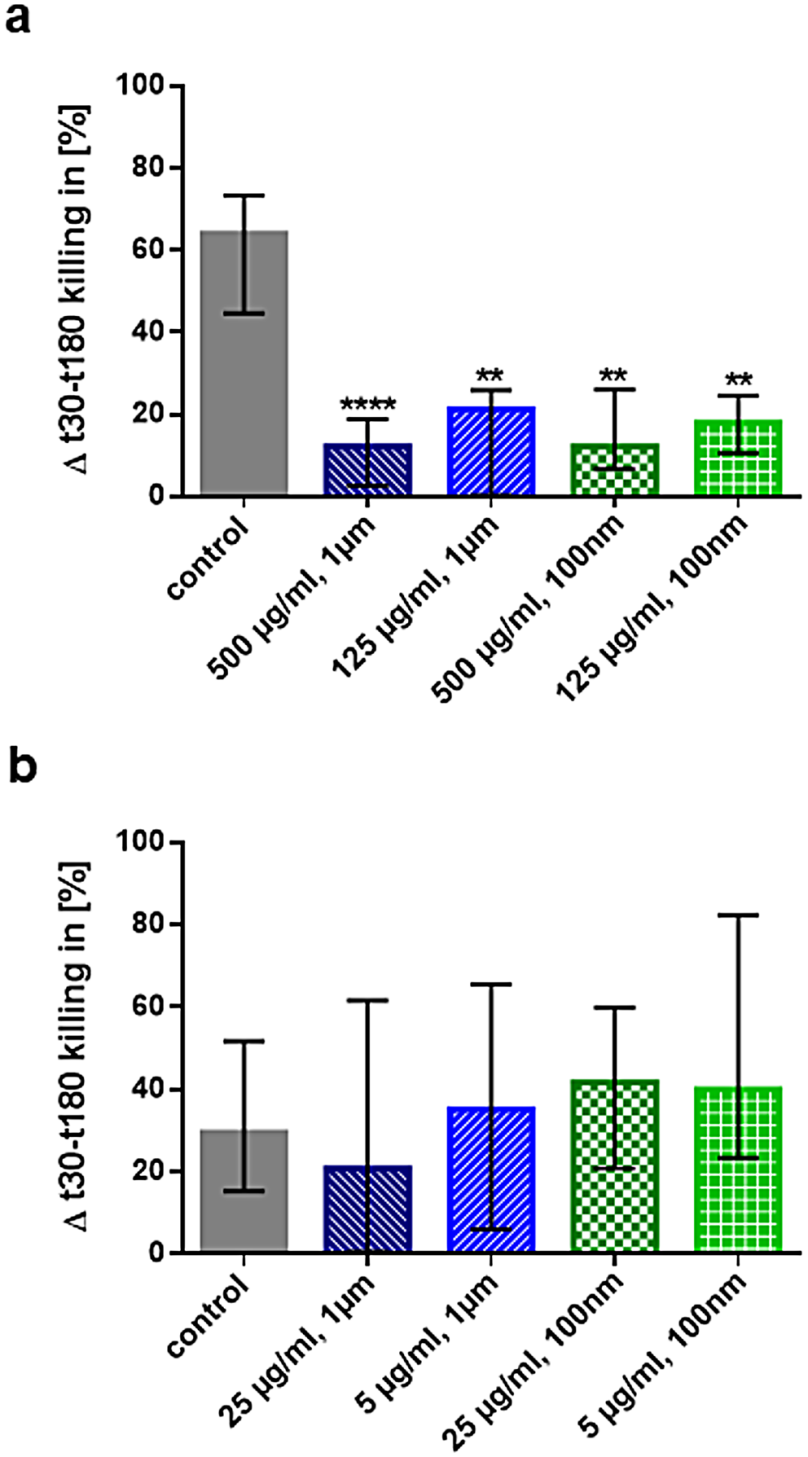
Micro- (1 μm) and nanoplastic (100 nm) at high concentrations inhibited intracellular killing of *E. coli*, whereas plastic at low concentrations did not alter the intracellular killing capacity of monocytes. Intracellular killing of *E. coli* by THP1-Blue™ NFκB monocytes (**a**) after pre-treatment with high concentrations of micro- or nanoplastic particles of 2 diameters (100 nm and 1 μm, polystyrene, plain surface) for 24 h and (**b**) after pre-treatment with low concentrations of micro- or nanoplastic particles of 2 diameters (100 nm and 1 μm, polystyrene, plain surface) for 24 h. Gentamicin was added to kill extracellular *E. coli* after 30 min of co-incubation of *E. coli* and monocytes. Statistical comparisons of all groups versus unstimulated controls; *n* = 24, columns represent medians, error bars indicate 25th and 75th percentiles; **p ≤ 0.01, ****p ≤ 0.001

After exposure for 24 h, the micro- and nanoplastic particles employed inhibited intracellular killing of *E. coli* in a concentration-dependent manner. At high concentrations (500 and 125 µg/ml) (Fig. 4a), micro- and nanoplastic reduced the intracellular killing significantly compared to unstimulated controls, but no difference between control and plastic-treated samples was detectable at low concentrations (25 and 5 µg/ml) (Fig. 4b).

### No activation of NFκB by plain micro- and nanoplastic particles

**Fig. 5:**
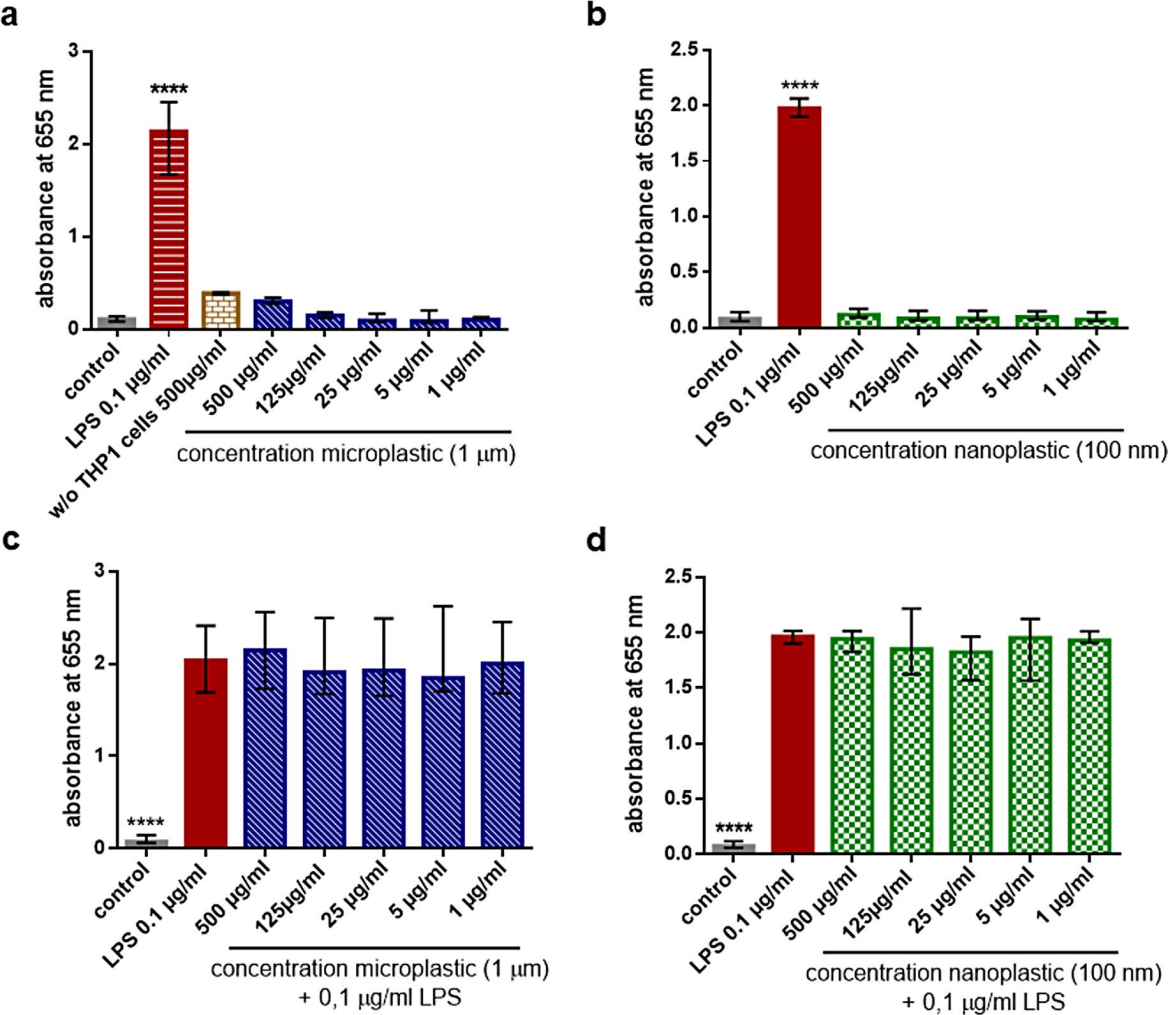
Absent activation of NFκB by plain plastic particles (**a, b**), and absent inhibition of the LPS-induced NFκB synthesis by plain plastic particles (**c, d**). In the transgenic human reporter cell line THP1-Blue™ NFκB, synthesis of NFκB induced activation of a secreted embryonic alkaline phosphatase (SEAP) reporter construct, which was quantified by the reagent QUANTI-Blue™. All concentrations of the plastic particles studied did not stimulate the pro-inflammatory NFκB pathway (**a, b**). Exposure to plain micro- (1 μm) and nanoplastic (100 nm) for 24 h did not alter the strong NFκB activation induced by LPS (**c & d**). Statistical comparisons of all groups versus unstimulated controls (**a, b**) or versus LPS 0.1 µg/ml (**c & d**); *n* = 15, columns represent medians, error bars indicate 25th and 75th percentiles; *p ≤ 0.05, **p ≤ 0.01, ***p ≤ 0.005, ****p ≤ 0.001

All concentrations of the plain plastic particles used did not stimulate the pro-inflammatory NFκB pathway (Fig. 5a & b). Exposure to plain micro- and nanoplastic for 24 h did not alter the strong NFκB activation induced by LPS (Fig. 5c & d). COOH-coated particles also did not activate NFκB at all concentrations tested. NH_2_-coated particles at concentrations > 25 µg/ml led to a mild concentration-dependent NFκB activation (Supplement, Figure S1).

### No substantial cytokine release and cytotoxicity after exposure to micro- and nanoplastic

After exposure for 24 h, the plain plastic particles used in the present study did not induce a substantial release of the pro-inflammatory cytokines IL1β [*n* = 18; plastic diameter 1 μm, concentration 500 µg/ml: median (25th /75th percentile) < 2 (< 2/<2) pg/ml] and IL6 [*n* = 21; <7.8 (< 7.8/34.5) pg/ml]. Stimulation for 24 h by LPS 0.1 µg/ml as positive controls led to an adequate release of IL1β [24.1 (23.0/32.0) pg/ml] and IL6 [125.3 (64.0/147.7) pg/ml] (Supplement, Figure S2). Compared to untreated cells, the micro- and nanoplastic particles at all concentrations tested (5–500 µg/ml) did not induce cytotoxic effects in THP1-Blue™ NFκB cells from 24 h to 120 h (Supplement, Figure S3 & S4). After exposure for 216 h, microplastic at concentrations ≥ 25 µg/ml had a mild dose-dependent cytotoxic effect (Supplement, Figure S4).

## Discussion

Our in-vitro study using a human monocyte cell line demonstrates that micro- and nanoplastic can affect phagocytosis and intracellular killing of bacteria in a time- and concentration-dependent manner. Moreover, micro- and nanoplastic particles at high concentrations were able to antagonize the stimulatory effect of LPS on phagocytosis. Previously, other effects of plastic particles on monocytes and macrophages have been described: in murine macrophages, the phagocytosis of microplastic induced a metabolic shift toward glycolysis and a reduction of mitochondrial respiration associated with an increased expression of the cell surface markers CD80 and CD86 and of cytokines associated with glycolysis [24].In the human THP1 cell line, after exposure to polypropylene microparticles of different size (0.5–3 μm) at concentrations ≥ 150 µg/ml and exposure durations ≥ 24 h, a concentration- and time-dependent increase of necrotic cells and ROS production were observed [25]. Polystyrene particles (size 20 nm – 1 μm) coated with carboxyl groups induced release of the cytokines IL-6 and IL-8, reduced phagocytosis of inactivated fluorescent *E. coli*, increased nitric oxide generation, myeloperoxidase release and oxidative burst and at high concentrations were cytotoxic in different assays using human whole blood, granulocytes or monocytes or murine macrophages [26]. In the absence of cytotoxicity, 0.5 and 1 μm carboxylated particles influence phagocyte function to a greater extent than smaller particles [26].

Our results show that 1 μm and 100 nm plastic particles inhibit phagocytosis and killing of live bacteria and illuminate the potential risks of the increasing pollution by small plastic particles on men`s and animals` immune defense. They suggest that all efforts possible should be undertaken to minimize further environmental pollution with these agents. Plain and COOH-coated plastic particles used in the present experiments at all concentrations tested did not induce an activation of the pro-inflammatory NFκB pathway. Unlike in a previous report using carboxylated polystyrene particles [26], pro-inflammatory cytokines were not released by plain micro- and nanoplastic in the present study suggesting that these particles do not induce a proinflammatory state. Because of the absence of inflammation, we hypothesize that the underlying mechanism of the inhibition of bacterial phagocytosis by plain plastic particles is overburdening of the phagolysosomal pathway possibly leading to accelerated aging as has been described for urban particulate matter [27]. A similar phenomenon has been observed during the removal of apoptotic cells. Macrophages carrying a high corpse burden were compromised in clearing wound debris [28]. At present, we are unable to prove this hypothesis. Future work will explore lysosomal dysfunction markers (e.g., cathepsin activity, lysosomal pH) [29] to support it.

The plastic concentrations used in the present study were high compared to concentrations measured in the environment. In seafood, plastic particle content was 0 − 10.5 particles/g in mollusks, 0.1 − 8.6 particles/g in crustaceans, 0 − 2.9 particles/g in fish, and 1 particle/g in echinodermata. Maximum annual uptake of plastic particles by humans was estimated close to 55,000 particles [10]. As a consequence of the applied methods, small particles might have been underestimated. Relatively high concentrations of plastic were measured in house dust from several countries [China (*n* = 39), Colombia (*n* = 45), Greece (*n* = 26), India (*n* = 33), Japan (*n* = 5), Kuwait (*n* = 18), Pakistan (*n* = 25), Romania (*n* = 21), Saudi Arabia (*n* = 30), South Korea (*n* = 16), the USA (*n* = 10), and Vietnam (*n* = 18)] ranging from 38 to 120,000 µg/g (median = 5900 µg/g) polyethylene and < 0.11–1700 µg/g (median = 8.8 µg/g) polycarbonate [3]. Comparisons of concentrations among different studies are difficult, because many studies report the number of particles instead of weight per volume, and the methods used have different minimum detectable particle sizes [3]. The highest plastic concentrations used by us (500 µg/ml) contained 0.95 × 10^9^ (particle size 1 μm) and 0.95 × 10^12^ (particle size 100 nm) particles/ml. These particle concentrations were several orders of magnitude higher than those measured in air outdoor and indoor even in large heavily industrialized cities, seafood including mussels and clams, tap and bottled drinking water and other beverages in plastic bottles [3]. In human whole blood from 22 healthy volunteers, the mean of the sum quantifiable concentration of plastic particles ≥ 700 nm measured by pyrolysis-gas chromatography/mass spectroscopy (PY-GC/MS) was 1.6 µg/ml [30]. Plastic particles with a size < 700 nm were not included in this analysis [30]. Therefore, the true micro- and nanoplastic concentration in the blood of healthy persons probably is higher and comes close to the lower concentrations (5 and 25 µg/ml) used in the present study. Long-term exposure by these plastic concentrations (9 days) affected phagocytosis of bacteria (Fig. 2b).

Under the assumption that 1 g of tissue has an approximate volume of 1 ml, the highest plastic concentrations employed by us (500 µg/ml) were in the same range as the total micro- and nanoplastic concentrations detected by PY-GC/MS in liver and kidney samples of autopsy cases (medians 433 and 404 µg/g in samples from 2024) and considerably lower than the concentrations measured in the brain tissue of persons without dementia (median 4917 µg/g in 2024) [31]. These recently published tissue concentrations suggest that human tissue macrophages may indeed be exposed to plastic concentrations corresponding to the highest concentrations employed by us. In demented patients, even higher concentrations of plastic were measured [31]. Nihart and co-workers demonstrated a significant increase of the plastic content in liver and brain samples from 2016 to 2024. This observation in conjunction with the undamped release of plastic into the environment makes a further increase of the plastic concentration in human tissues likely and explains lower concentrations measured in previous human studies (placenta: median = 63.4 µg/g; testes: median = 299 µg/g) [32, 33]. To sum up, concentrations in heavily contaminated environmental material such as house dust and human tissue concentrations were in the same range as the highest concentrations studied by us. Here, plastic concentrations of 500 µg/ml impaired phagocytosis within 24 h. The inhibitory action of plastic particles on bacterial phagocytosis increased with time. As a consequence of the limited durability of the cell cultures used, we were unable to study cells for more than 216 h (= 9 days). In real life, the exposure times are much longer (potentially life-long) probably increasing the effect of small plastic concentrations on the immune system. Therefore, our observation most probably is of physiological relevance.

The major strength of this study is the use of live instead of dead bacteria and of a human cell line instead of monocyte or macrophage cultures from experimental animals. This allowed us to construct concentration- and time-response curves without inter-person variations and facilitates the transfer of the results to humans. The use of transgenic THP1-Blue™ NFκB cells is a sensitive method to detect an inflammatory response, which in the present study with plain and COOH-coated particles was absent. Consequently, we were unable to detect a substantial release of the major pro-inflammatory cytokines IL-1β and IL-6 by plastic particles. Another strength is the use of defined plain plastic particles as stimulants avoiding inter-experiment variation. This strength of our study at the same time is its most severe limitation: the results obtained with plain polystyrene particles cannot be generalized to other plastic types or environmentally degraded particles. In the present project, plain polystyrene particles were rapidly internalized by THP1-Blue™ NFκB monocytes. This was not accompanied by an inflammatory response as illustrated by the absence of NFκB activation and of the release of pro-inflammatory cytokines. In previous studies, the surface of micro- and nanoplastic particles of similar magnitude influenced their uptake into the mammalian body and the penetration through cell membranes including the entry into the CNS. Polystyrene coated with NH_2_ and (to a smaller extent COOH) residues penetrated cell membranes more readily than plain polystyrene particles [34, 35]. The inflammatory activity and toxicity of micro- and nanoplastic particles is modulated by molecules, including proteins, lipids or polysaccharides, which adhere to their surface after contact with biological fluids. These adhering molecules may substantially alter the physical and chemical properties of microplastic particles including their effective size, electric charge, hydrophobicity and immunostimulatory activity [36]. Here, plain and COOH-coated microplastic particles did not activate the transcription factor NFκB. Conversely, NH_2_-coated microplastic particles led to a mild concentration-dependent NFκB activation (Figure S1). Alteration of the plastic surface by weathering may further increase the toxic and proinflammatory activity of plastic particles generated under natural conditions on eukaryotic organisms compared to uniform plain plastic particles used in the present study [37]. For these reasons, the effect of plain plastic particles on eukaryotic immune cells observed here probably represents the minimum of inflammation which can be expected after exposure of human immune cells to plastic particles. The other limitation of our study is the use of a cell line instead of primary human cells. The THP1 cell line is known to produce lysozyme and to phagocytose [20] and thus possesses many features of primary macrophages and monocytes. It may – however – not secrete all cyto- and chemokines released by primary cells upon the stimuli employed [38]. Moreover, THP1 cells stimulated with LPS produce approx. one order of magnitude lower proinflammatory cytokine concentrations than equal numbers of primary human monocytes [39]. For this reason, complementary studies with primary cells or whole-blood assays are planned. The use of primary human blood monocytes or granulocytes or of a human whole blood assay probably will introduce a large interindividual variation [23], which will require a high number of volunteers. It is also planned to study the impact of plastic particles with physiological alterations of their surface, e.g. by weathering, on phagocytosis and intracellular killing of bacteria.

In conclusion, polystyrene plastic particles at concentrations detected in house dust and some human tissues inhibited phagocytosis and intracellular killing after exposure of 24 h to a human monocyte cell line. With lower concentrations of plastic particles, co-incubation of several days was necessary to inhibit bacterial phagocytosis. Plain particles did not cause inflammation as evidenced by absent NFκB synthesis and proinflammatory cytokine release and did not induce LDH release as a surrogate of cell death. Since micro- and nanoplastic from natural sources have more complex surface structures, they may have a stronger impact on bacterial phagocytosis than the particles studied here. Our results stress the necessity to reduce the release of plastic into our environment to protect our immune system. A continuation of “business as usual” will triple the plastic production and waste generation by 2060. The Organization for Economic Cooperation and Development (OECD) developed strategies to reduce the dispersal of plastic waste to aquatic environments. Yet, even in ambitious policy scenarios, a peak in land to sea transport of total plastics will be reached approx. 2045, and environmental concentrations of microplastic and nanoplastic particles will remain high after 2060 [40].

## Electronic supplementary material

Below is the link to the electronic supplementary material.

Supplementary Material 1

## Data Availability

No datasets were generated or analysed during the current study.
