# Supplementary material for: Micro- and nanoplastics reduce the phagocytosis and intracellular killing of E. coli by THP1-Blue™ NFκB monocytes
Source: Infection. 2025 May 26;53(5):2179–89. doi: 10.1007/s15010-025-02565-1 (PMC12460519; doi:10.1007/s15010-025-02565-1)
Supplement: Supplementary file 1 — Supplementary Material 1 [file 15010_2025_2565_MOESM1_ESM.docx]

**Supplementary Figures**

**Micro- and nanoplastics reduce the phagocytosis and intracellular killing of *E. coli* by THP1-Blue^TM^ NF**κ**B monocytes**

*Florian Edbauer^1^, Hans-Christoph Ludwig^1^, Marie Julia Moritz^1^, Roland Nau^1,2^, Jana Seele^1,2^*

^1^Department of Neuropathology, University Medical Center Göttingen, Georg-August-University Göttingen, Göttingen, Germany; ^2^Department of Geriatrics, Evangelisches Krankenhaus Göttingen-Weende, Göttingen, Germany

E-mail of the corresponding author: rnau@gwdg.de


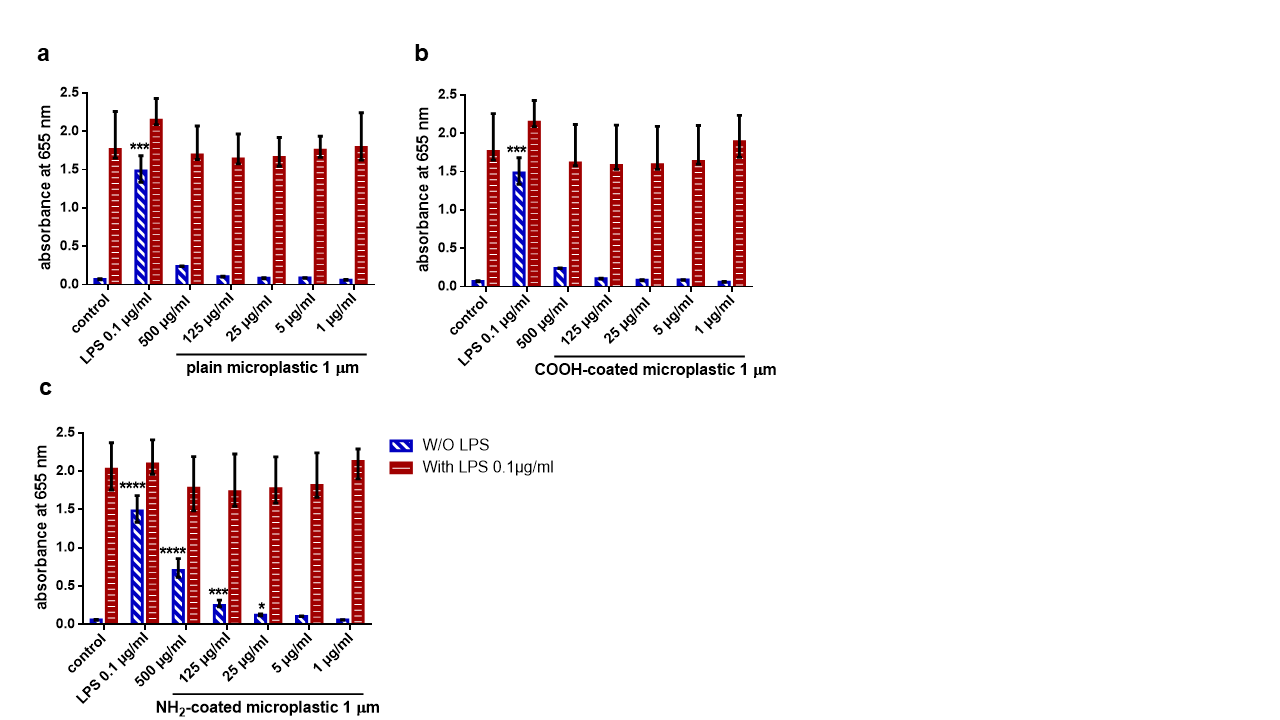


Figure S1: Activation of NFκB in THP1-Blue^TM^ NFκB monocytes by LPS 0.1 μg/ml after pre-treatment with microplastic (polystyrene, diameter 1µm) at different concentrations for 24h

a) plastic particles with plain surface, b) COOH-coated plastic particles, c) NH_2_-coated plastic particles.

Plastic particles with plain surface and COOH-coated particles did not activate NFκB at all concentrations tested. NH_2_-coated particles at concentrations >25 μg/ml led to a mild concentration-dependent NFκB activation. All types of plastic particles did not inhibit the subsequent NFkB activation by LPS. Statistical comparisons of all groups versus unstimulated controls; each group n = 12, columns represent medians, error bars indicate 25^th^ and 75^th^ percentiles; *p < 0.05, **p ≤ 0.01, ***p ≤ 0.005, ****p ≤ 0.001.


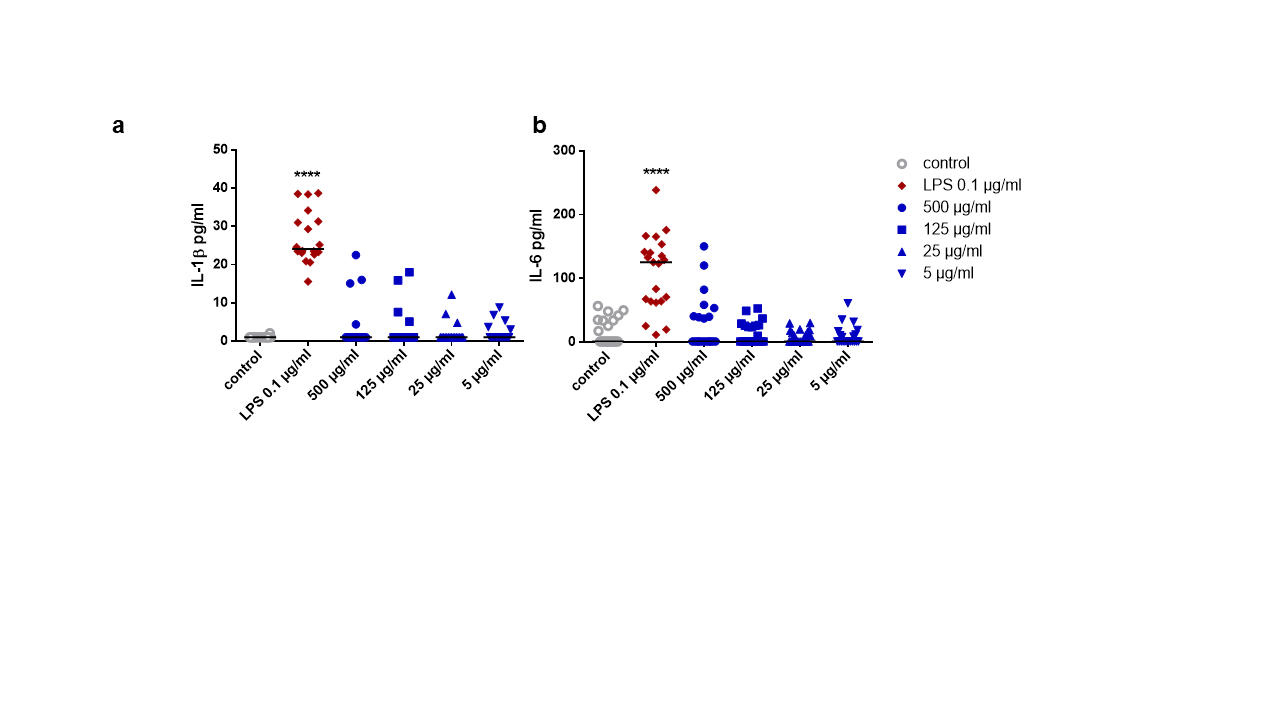
Figure S2: Cytokine release by THP1-Blue^TM^ NFκB monocytes after pre-treatment with LPS 0.1 μg/ml or microplastic (polystyrene, plain surface, diameter 1µm) at different concentrations for 24h. a) Interleukin (IL)-1β (n = 18); b) IL-6 (n = 21). Horizontal bars represent medians, symbols indicate individual measurements. Please note that only LPS induced a robust release of IL-1β and IL-6 (****p < 0.001 versus unstimulated controls). Plain microplastic did not induce a significant release of IL-1β or IL-6 (Kruskal-Wallis test followed by two-tailed Dunn’s multiple comparisons test to correct for repeated testing).


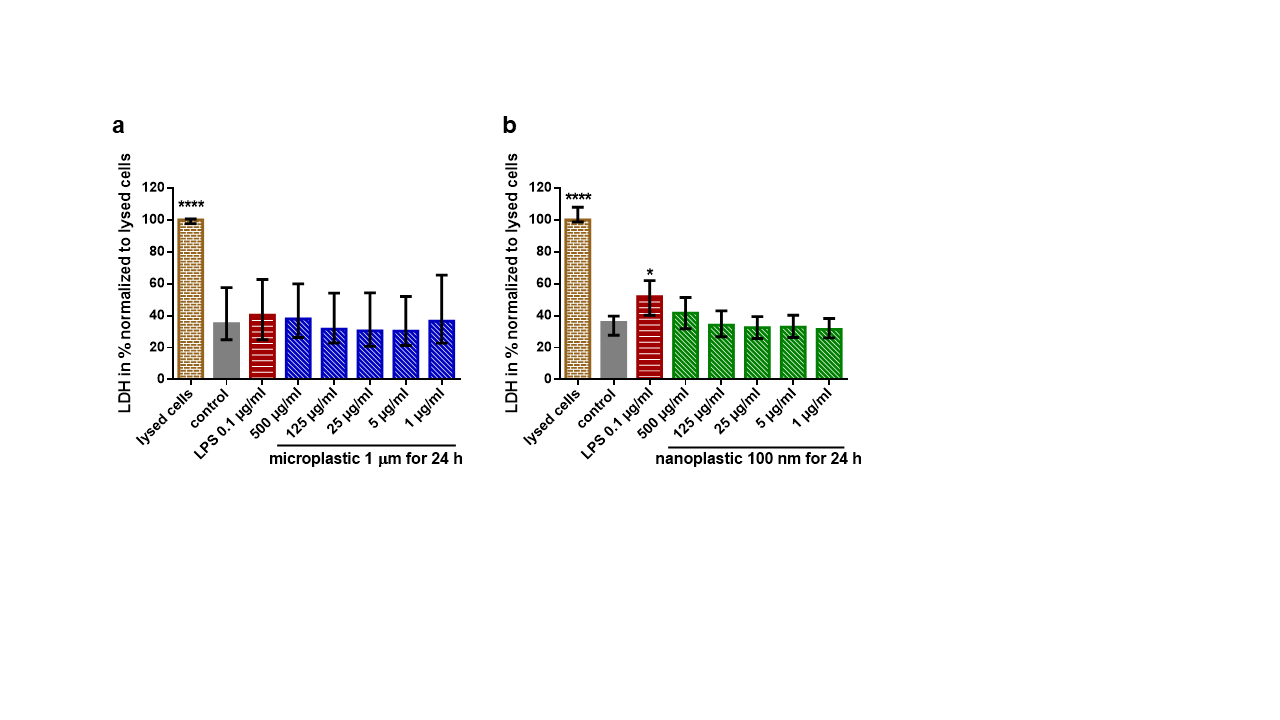


Figure S3: Lactate dehydrogenase (LDH) release by THP1-Blue^TM^ NFκB monocytes after pre-treatment with LPS 0.1 μg/ml or a) microplastic (polystyrene, plain surface, diameter 1µm) or b) nanoplastic (polystyrene, plain surface, diameter 100nm) at different concentrations for 24h (n = 12, columns represent medians, error bars indicate 25^th^ and 75^th^ percentiles). Please note that neither micro- nor nanoplastic caused a substantial LDH release compared to unexposed control cells (lysed cells versus all other groups p < 0.001). LPS stimulation caused a mild LDH release (p < 0.05 versus untreated controls in b). Kruskal-Wallis test followed by two-tailed Dunn’s multiple comparisons test to correct for repeated testing.


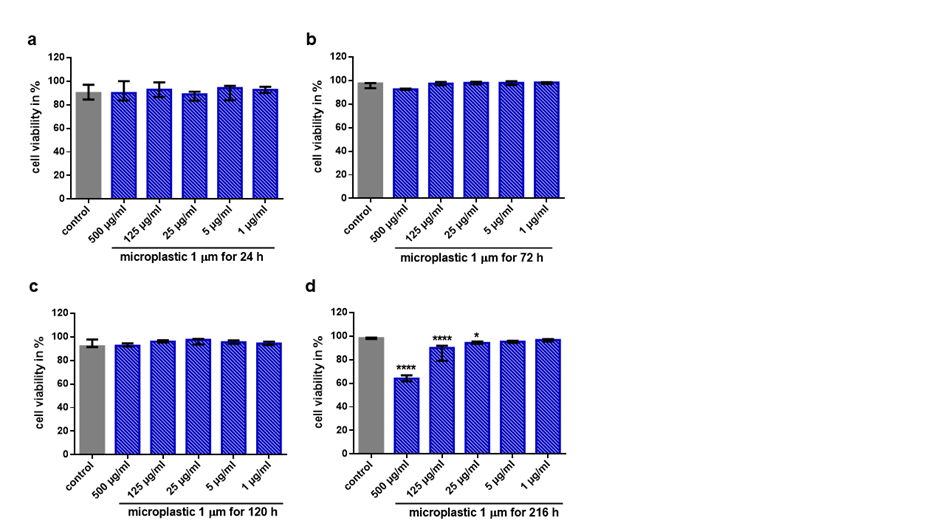


Figure S4: Cell viability of THP1-Blue^TM^ NFκB monocytes assessed by trypan blue exclusion after exposure to different microplastic (polystyrene, plain surface, diameter 1µm) concentrations for 24h, 72h, 120h or 216h. From 24h to 120h, no cytotoxicity of the plastic particles was observed. After exposure for 216h, microplastic at concentrations ≥25 μg/ml had a mild dose-dependent cytotoxic effect (*p < 0.05, ****p < 0.001.; Kruskal-Wallis test followed by two-tailed Dunn’s multiple comparisons test to correct for repeated testing).
